# Supplementary material for: Delivering effective, comprehensive, multi-exercise component cardiac rehabilitation (CR) for chronic heart failure patients in low resource settings in sub-Saharan Africa: Queen Elizabeth Central Hospital—(QECH-CR) randomised CR study, Malawi
Source: PLoS One. 2024 May 24;19(5):e0297564. doi: 10.1371/journal.pone.0297564 (PMC11125511; doi:10.1371/journal.pone.0297564)
Supplement: S1 File — (PDF) [file pone.0297564.s003.pdf]

COLLEGE OF MEDICINE RESEARCH AND ETHICS COMMITTEE

Request for Amendment/Modification

Please complete the following:

**COMREC REF. Number**<br>P.10/20/3167<br><br>(COMREC will not process requests without this number.)	**Date of Request**<br>4th June, 2021
**Principal Investigator Name: Alice Namanja**<br><br>Phone # : 0997759408 Email: anamanja@medcol.mw	**Contact Person** (if other than PI)<br><br>**Phone #** **Email**
Title of Study<br>Impact of Cardiac Rehabilitation on cardiorespiratory parameters: Towards establishment of CR intervention at Queen Elizabeth Central Hospital	

1. **Description of proposed changes:** We Intend to use Chester step test for exercise prescription and maintain 6-minute walk test just for exercise tolerance capacity. We also intend to add a general patient assessment form. Furthermore, we have added a checklist that will be used by Physiotherapists to report challenges they may encounter in the project from enrolment, intervention delivery to follow up after completion of the intervention delivery. Lastly we have added a weekly vital signs recording sheet

2. **Reason for Amendment/Modification:** We intend to use Chester step test for exercise prescription because the test procedure does not require more than 1 person to conduct the test unlike the 6-minute walk test for accurate results. We wish to maintain standards and ensure quality and reliable outcomes, which we will safely use to prescribe an effective intervention. Therefore, we have added a Chester step test checklist to the list of appendices.

   We also intend to add a general patient assessment, which will be used to assess patients enrolled at baseline and capture all risk factors for an appropriate and adequate planning of the education and counselling therapies. This is not included in the approved protocol, but it is essential to be included.

   We intend to add a checklist for Physiotherapists because this would also assist us in understanding the practical or technical and operational challenges we may face in delivering this intervention as health care workers. We need to understand the challenges from both patients and Physiotherapists. This would assist us to answer the

third specific objective. The weekly vital signs recording sheet will assist us to ensure that we monitor haemodynamic parameters of the participants on each day of therapy.

3. **Changes to Consent Form:** Are changes required? **Yes**
(If Yes, attach new consent form)

Principal Investigator signature	Date

COMREC Office Use only:
Approval date:____________________

Approved by:
____

Approval of Changes /Modifications by COMREC

Signature **Date**

Recommended :_______
Not recommended :_______

IRB Chairperson or Authorized Signatory

____

COMREC form 103

Address: College of Medicine Research and Ethics Committee / Mahatma Gandhi Road, Chimutu Building Room # 822, P/Bag 360 Chichiri, Blantyre 3 Telephone: (265) 01 871 911/01 874 377Fax (265) - 01 874 740 **E-mail:** comrec@medcol.mw

Version: #1:0 **Effective date: 25th November, 2007**

UNIVERSITY OF MALAWI - COLLEGE OF MEDICINE RESEARCH and ETHICS COMMITTEE (COMREC) CHECKLIST TO ACCOMPANY NEW RESEARCH PROPOSALS SUBMITTED TO THE COMMITTEE

*When you submit a research proposal for the Committee to approve, first read the document entitled **COMREC Elements Of Review** attached to this check list. Make sure that your proposal is in the format outlined in the document.*

Before sending or giving the proposal to the Committee, complete the following check-list by ticking each item you have included. Do not submit the proposal *unless you can tick all the boxes, or provide a reason for the absence of any item. Attach the completed check-list to the front of your submission. Provide evidence of payment of US150 processing fee.*

TITLE OF PROPOSAL: **Impact of Cardiac Rehabilitation on cardiorespiratory parameters: Towards establishment of CR intervention at Queen Elizabeth Central Hospital**
**Name of Principal Investigator:** Alice Namanja
**Name of Sponsor and amount of sponsorship:** NCD-BRITE, MK10,516,022

Submit all documents in one pdf file of not more than 5MB by email to comrec@medcol.mw (if the file size is more than 5MB, then please zip the file and submit it as a compressed zipped file). The **single** pdf file should include the following information in the following order:

01	Completed copy of this checklist as stated above		Yes☒or No☐
02	Covering letter of introduction from Investigator		Yes☒or No☐
03	The study protocol which should include the following:-		
	Study Title		Yes☒or No☐
	List of Investigators and institution(s) involved		Yes☒or No☐
	Executive Summary		Yes☒or No☐
	Background/Introduction		Yes☒or No☐
	Rational/justification		Yes☒or No☐
	Objectives of the study: Main objective and Specific Objectives		Yes☒or No☐
	Methods:		
		Type of study - place of study	Yes☒or No☐
		Study population	Yes☒or No☐
		Study period Sample size	Yes☒or No☐

		Data collection procedures	Yes☒or No☐
		Data management/analysis	Yes☒or No☐
		Presentation of results	Yes☒or No☐
	Dissemination of results		Yes☒or No☐

	Ethical considerations: including consenting procedures, participant compensation, participant confidentiality etc.	Yes☒or No☐
	Possible constraints	Yes☒or No☐
	Requirements	Yes☒or No☐

	Training provided for study staff	Yes☒or No☐
	Budget and Justification of budget	Yes☒or No☐
	References	Yes☒or No☐
04	Consent forms: include consent forms in both English & Chichewa for adult participants aged 18 and above, parental consent forms for all minors and assent forms (in addition to the parental consent forms) for all minors between the ages of 7 and 17 years. Any participant payments e.g. compensation, reimbursement etc should be stated by amount in the consent forms.	Yes☒or No☐
05	Data collection tools (proformas): those that will involve obtaining information from research participants should be translated into Chichewa	Yes☒or No☐
06	Material transfer agreement forms/documents	Yes☒or No☐
07	Have you applied for a waiver of 10% COM overhead fee from the Office of Postgraduate Dean of Studies and Research? If yes, please attach a waiver letter.	Yes☐or No☒
08	Have you submitted this proposal to another Ethics Committee? If yes, please specify whether approval has been given, and if approval has been awarded, please submit a copy of the approval letter with this submission	Yes☐or No☒
09	Letter of support from COM Head of the Principal Department hosting the research	Yes☒or No☐
10	Letter(s) of support from Heads of all other Depts. and institutions in which any research work will be done.	Yes☒or No☐
11	Evidence of current active registration with the Medical Council of Malawi for Principal Investigator and other investigators who are involved in clinical research	Yes☒or No☐
12	Brief CV of each investigator	Yes☒or No☐

If any item is not ticked, explain why this is not included with the submission.

Signed: Name (print): Alice Namanja

Date:10/03/2021

Principal

Mac Mallewa, MBBS, PhD.

College of Medicine

Private Bag 360

Chichiri

Blantyre 3
Malawi

Your Ref:

Our Ref: P.10/20/3167

Telephone: 01871911
01874107

Fax: 01 874 700

The chairperson
College of Medicine Research and Ethics Committee

Dear Sir,

SEEKING FOR AMMENDMENT

I write to seek for amendments to my protocol for a study titled "impact of cardiac rehabilitation on cardiorespiratory parameters; Piloting towards establishment of CR intervention at

Queen Elizabeth Central hospital", which is being funded by NCD-BRITE. I have added some tools, which were mentioned in the protocol but were not added as appendices, with just one new tool added to the protocol (number 2 on the list below). I have also added one statistical test, independent t-test, which was missed but essential for this controlled study. The additions are as follows;

1. Added independent t-test in the data analysis plan to compare the results between the two groups.
2. Added Chester step test for exercise prescription and maintained use of 6-minute walk test for exercise tolerance capacity (Appendix 9).
3. Added a general patient assessment form as Appendix 10.

4. Added a checklist that will be used by Physiotherapists to report challenges they may encounter in the project from enrolment, intervention delivery to follow up after completion of the intervention delivery. (Appendix 11)
5. Added a weekly vital signs recording sheet as an appendix12

All the new additions are coloured in yellow for easy tracking.

Looking forward to hearing from you soon.

Alice Namanja.

Impact of Cardiac Rehabilitation on cardiorespiratory parameters: A pilot study towards establishment of CR intervention at Queen Elizabeth Central Hospital

Investigator: Alice Namanja

Physiotherapist and Lecturer, University of Malawi- College of Medicine

Mentor: Professor Johnstone Kumwenda

Physician and Professor, University of Malawi- College of Medicine and John Hopkins research Centre

Co-mentor: Dietmar Seidenspinner

Physiotherapy lecturer and Integrated expert advisor (GIZ)

Executive summary

Background and problem: The burden of cardiovascular disease (CVD) in low and middle income countries is expected to double by 2030. In 2014, 12% of deaths attributable to non-communicable diseases (NCDs) were due to CVD. Congestive Heart failure (CHF) is one of the common CVDs resulting in patient admission at the Queen Elizabeth Central Hospital (QECH). While medications for the treatment of CHF are readily available at the hospital, holistic management of this syndrome is lacking. One important facet of holistic management is cardiac rehabilitation after a diagnosis of severe congested cardiac failure. We plan to carry out a pilot study to determine the impact of cardiovascular rehabilitation among patients being discharged from the QECH with a diagnosis of CHF.

Specific Objectives:

1. To describe improvements in cardiovascular function at rest and on exercise among patients with a diagnosis of CHF
2. To describe the frequency of hospitalization among patients diagnosed with CHF enrolled into the cardiovascular rehabilitation program (CRP).
3. To describe the challenges that may be associated with establishment of a CRP at the QECH.

Study design, population, sample size and sampling strategy: This will be a prospective open label clinical trial of patients being discharged from the medical wards at QECH with a diagnosis of CHF. There will be two arms in the study with a total of 80 participants; Arm 1 will comprise of 40 patients with CHF who will receive cardiovascular rehabilitation as an intervention of interest, along with the usual cardiac medications that also include secondary prevention drugs, while 40 patients with CHF in arm 2 will be on cardiac medications that also include secondary prevention medications and education but not the exercise component of cardiac rehabilitation. Each participant will pick unlabelled sealed envelope with a paper that has a number that will determine to which group he or she will belong to. Participants will be followed up for 24 weeks. Participants in arm 1 will have regular cardiovascular exercise training once a week for 12 weeks and followed up without active exercise training for another 12 weeks. Arm 2 participants shall receive education component of cardiac rehabilitation at baseline and during the time points they will come for screening. Participants in both arms 1 and 2 will

continue to take cardiac medications as per their cardiologist's prescription throughout the study period.

Inclusion criteria

a. Men and women aged 18 years and above with a diagnosis of CHF, confirmed with an echocardiogram, at discharge from hospital
b. Being residents of Blantyre City and its immediate surrounding areas
c. Willing to provide written informed consent and willing to return for follow up
d. Being able to participate in physical activity for cardiac rehabilitation

Exclusion criteria

a. Failure to provide informed consent form
b. Not willing to return for follow up visits
c. Physical impairment that would affect participation in physical activity
d. Coming from outside Blantyre
e. Patients classified as high risk for cardiac events during exertion

Data management and analysis**:** Descriptive analysis will be conducted using SPSS software; frequencies and mean changes with standard deviations and confidence intervals will be used to report the effect size of the intervention. Multivariate paired T tests will be conducted to compare the changes in each variable at the five time points, and independent t-test will be conducted to compare the changes between the two groups. Tables and graphs will be used to present the findings**.**

Expected outcomes: Participants in arm 1 will have better cardiovascular function and fewer hospital admission compared with the controls during the first 12 weeks and that these benefits will remain at 24 weeks.

Dissemination plans: Findings from the study will be presented to QECH management and clinicians involved in managing patients with CHF. The findings will also be presented at the COMREC dissemination conference. Furthermore, the results will be submitted to a peer review journal for publication.

Anticipated challenges: It is anticipated that patients may be lost to follow up and discontinue with the prescribed intervention due to transportation related costs. Therefore, the patients enrolled in this program will be assisted with the transportation costs; patients enrolled will be those who will manage to travel the distance with the allowance given. In addition, patients will be asked to come for a therapy session at the hospital once each week and do the other two sessions at home with a trained family member.

1.0 Background information and introduction

Malawi continues to fight against Cardiovascular Disease (CVD), a disease that claimed 18 million lives globally in 2016 (1). Cardiovascular disease is a term that encompasses all diseases and disorders of the heart and blood vessels (2). Sadly, non-communicable diseases accounted for 28% of total deaths in Malawi in 2014, of which 12% was attributed to CVD (3) . Although the risk of dying due to CVDs, in adults aged 30-70-years, reduced globally from 22% in 2000 to 18% in 2016, the WHO (1) reported that the burden of CVDs in developing countries is on the rise and is projected to increase further by 2-fold (4). Furthermore, it is known that the pattern of cardiac disease varies in different countries and factors such as race, age and economic status of the nation's attribute to this variation (5).

For instance, a descriptive study done in the past five decades at Queen Elizabeth Central hospital (QECH) showed that a larger proportion (23%) of 114 cardiac patients had rheumatic heart disease (6). Remarkably, patients found with this condition were under 20 years of age (6); suggesting possible existence of common causal factors in this age group. Although only 16% of the patients had hypertensive heart disease, it was found that a larger proportion of this group (87%) were aged

40 and above (6); this implies that this heart disease was common in patients over the age of 40 in this population. Furthermore, a few individuals had TB (7%) and pulmonary (8%) related heart failure (6). Nevertheless, this study involved doing electrocardiography, chest x-ray imaging analysis and clinical presentation of patients. Additionally, HIV pandemic was not in existence then. Therefore, such cardiac disease categories may not be generalized to entire Malawian population. However, a recent study at the same site used echocardiogram and found that most of the 117 cardiac patients (44%) had hypertensive heart disease, seconded by dilated left cardiomyopathy (18%), TB related pericardial effusion (14%) and only 9% accounted for rheumatic heart disease (7). Although it is reasonable to postulate that there has been a change in the pattern of the most occurring heart disease in Malawi from rheumatic to hypertensive heart disease over the past four decades, it should be noted that the latter study had a mean age of 47 years and most patients were HIV positive (37%) or had their status unknown (21%) (7). This further suggests that hypertensive heart disease is associated with aging, and HIV infection might have attributed to the rising cases of heart disease in the country.

Correspondingly, Allain (8) conducted echocardiograms on over 800 cardiac patients at QECH over a period of 6 years and found that 20%, 19%, 12% and 9% of cases accounted for left ventricular failure, pericardial disease, valvular heart disease and right heart failure respectively. Surprisingly, only 7.5% and 1% of the population had hypertensive and ischaemic heart disease respectively (8). Similar findings were reported by Soliman and Juma (9); they found that less than 1% of 3908 cardiac patients at Mzuzu Central Hospital had coronary artery disease; suggesting a least occurring cardiac pathology in a Malawian population. Soliman and Juma (9) reviewed a cardiac clinical registry book of all patients that were seen in a period of 5 years at Mzuzu Central Hospital, which showed that the majority (34%) had rheumatic heart disease, seconded by hypertensive heart disease (24%), cardiomyopathy (19%) and pericardial disease (14%). Although the age ranged from 2-82 years in the aforementioned study, this suggests that rheumatic and hypertensive heart diseases are the most occurring cardiac conditions in Malawi.

In other sub-Sahara African countries, the cardiac disease pattern seems similar to the changing trend in Malawi. Between 2006 and 2010, South Africa and Nigeria had hypertensive heart disease as the most occurring cardiac disease among the cardiac patients (41% and 61% respectively) (10) (11). Although Tanzania recorded a high prevalence of pericardial effusion (41%) between 2009 and 2010, hypertensive heart disease was also high (34%) (12). Furthermore, majority of cardiac patients in Botswana had cardiomyopathy (37%), pericardial disease (21%) and hypertensive heart disease (14%) in 2012 (13). Similar trend of CVD pattern was reported in Zimbabwe between 1994-1998 (14) (15). Although the latter proportion seems relatively small compared to the other two, this implies that the hypertensive heart disease is common in the African countries. Therefore, it is reasonable to postulate that hypertensive, rheumatic heart diseases and cardiomyopathy are the most common occurring cardiac disorders in SSA. This also implies that hypertension is becoming a leading cause of cardiac disorder among adult population in SSA while rheumatic heart disease continues to affect younger adults and children.

In Malawi, cardiac patients are put on appropriate medications once diagnosis is made, and when surgical need arises, patients are advised to seek for further medical help in other countries such as South Africa and India because the nation does not have the capacity to conduct heart surgeries. Nevertheless, Malawi does not offer a comprehensive package of CVD management as recommended by the WHO (16) because it does not incorporate Cardiac Rehabilitation (CR) in the CVD treatment protocol. CR is defined as a holistic approach, championed by patients themselves, deployed in efforts to (i) minimise or eliminate the main causative agents of CVD that could also rapidly progress the disease severity, and (ii) promote social, mental and physical wellbeing of the people; thereby promoting health-related quality of life and functional independency (17). This implies that the success of this intervention mainly depends on the willingness of a patient to participate and adhere to the management protocol and advice given. The CR is conservative and can easily be delivered in poor settings using the modified approaches as suggested by Grace et al. (18) and WHO (16). Figure 1 illustrates the core components of CR, which are delivered by various

specialists such as Physiotherapists, cardiologists, psychologists, nurses and pharmacists who work in a coordinated way to deliver the CR (17)(19).

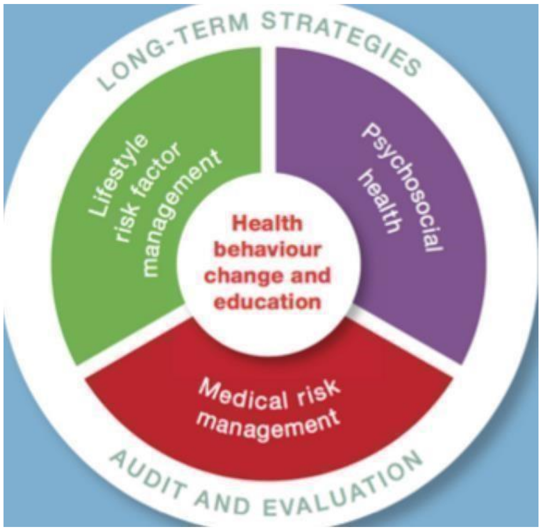

Figure 1. The main components of CR implementation (BACPR, 2017)

The core components serve as a backbone onto which a successful CR intervention emerges from.

For instance, the ‘lifestyle risk factor management’ component involves issues such as encouraging tobacco smoking cessation, healthy eating habits, weight management, exercises and physical activities (17). This suggests that acquisition of self-management skills is vital to enhancing cardiovascular health both at secondary and tertiary levels. Additionally, CR aims to effectively integrate CVD patients into their communities with reasonable functional capacity that would enable them to participate in carrying out day to day activities (20) (16).

For instance, a meta-analysis of clinical trials by Anderson and Taylor (21) showed that CR attributed to lowered cardiac related deaths (RR 0.74, 95%CI 0.64-0.86); suggesting that the intervention can potentially delay progression of the disease and lead to a prolonged life. Similarly, Long et al. (22) argued that exercise therapy embedded in the CR intervention improves maximal oxygen consumption in cardiac patients (Standard Mean Deviation [SMD] 0.45, 95% CI 0.200.70),

which further indicates the capacity of the intervention to prolong lives and improve functional capacity of cardiac patients. Furthermore, a recent experimental study by Choo et al. (23) suggested that a Cardiac Rehabilitation Program (CRP) significantly improved both physical and mental quality of life mean scores (Before 41.4 ±8, After 47.2±7; Before 50±8, after 52.5±6, respectively, All: p=0.001). Similarly, depression and anxiety mean scores reduced following a CRP (Before 4±3, After 2.5±2, p=0.001; Before 4.5±4, After 4±3, respectively) (23). Despite the aforementioned positive clinical health outcomes, Malawi has not championed CR intervention. It is also not known if implementation of this strategy would be beneficial to the cardiac population in Malawi.

2.0 Problem statement and justification

Although it is feasible to implement CRP in poorly resourced areas, Malawi has not championed the intervention. Additionally, it is known that the treatment guidelines of CVD in Malawi do not clearly incorporate CR as an integral component of the management process. Therefore, there is need to conduct a pilot CR intervention to inform the feasibility and importance of the strategy. The outcomes of this pilot program will also sensitize the healthcare providers and managers on the need to incorporate CRP in the CVD management guidelines for Malawi. In addition, the findings will inform the strategic planning and implementation of the CR intervention at QECH, which would be scaled up to other hospitals; thereby increasing accessibility.

3.0 Objectives

3.1 Broad objective

The overall aim of this study is to carry out a pilot study to determine the impact of cardiovascular rehabilitation among patients with a diagnosis of Congestive Heart Failure (CHF).

3.2 Specific Objectives

1. To describe the improvements in cardiovascular function among patients with CHF following administration of CR
2. To describe the frequency of hospitalization among patients diagnosed with CHF
3. To describe the challenges that may be associated with establishment of a CRP at QECH

4.0 Methodology

4.1 Design

The study shall use a prospective open label clinical trial design, and quantitative methods will be employed.

4.2 Study site

The study will be conducted at QECH, in Physiotherapy department

4.3 Study population

Patients with CHF, who come for follow up medical care at the QECH chest clinic at QECH, shall constitute the target population.

4.31 Inclusion criteria

a. Men and women 18 years and above with a diagnosis of CHF at discharge from hospital, confirmed with an echocardiogram
b. Being residents of Blantyre City and its immediate surrounding areas
c. Willing to provide written informed consent and willing to return for follow up
d. Being able to participate in physical activity for cardiac rehabilitation

4.32 Exclusion criteria

a. Failure to provide informed consent form
b. Not willing to return for follow up visits
c. Physical impairment that would affect participation in physical activity
d. Coming from outside Blantyre
e. Patients classified as high risk for cardiac events during exertion

4.4 Sample size, sampling method and study period

This study will enrol patients with CCF, who will come for medical check-up during the eight-week period of study participants' enrolment. According to the medical register book, there is a varying number of CCF patients who come for the clinic each week. For instance, out of a total of 53, 212 and 130 patients who came to receive cardiac or respiratory related medical care through this chest clinic in January, February and March, 19, 45 and 28 patients had CCF respectively. The clinic was temporarily shut down between April and September due to COVID-19 pandemic, and it recently resumed its services. However, out of 77 and 116 patients who came to this chest clinic in October and November after resumption of the clinic's services this year, 5 and 13 patients had CCF. This shows a drastic reduction in the number of cases coming for medical care; nevertheless, the numbers have reduced as a strategy to avoid congestion in the clinic and protect the patients from contracting or spreading the SARS-2 virus that causes COVID-19. It is expected that the number of patients coming for this clinic will remain low until Malawi is declared COVID-19 free or further measures have been put in place to protect patients at this clinic. Therefore, the following formula has been used to calculate the sample size with reference to a similar study conducted by
Awotidebe et al. (2016) in Nigeria and the trend of cardiac patients attending the chest clinic at QECH;

$$n = 2 \, \frac{[(Z_{\alpha}+Z_{\beta}) \quad x \quad 2(\sigma)^2]}{(\mu_1-\mu_2)^2}$$

Where μ_1=mean in group 1, μ_2=mean in group 2, $Z\alpha$=0.9, $Z\beta$=0.84 (power), σ=1.2

The sample size for this study is based on detecting the effect size (the difference between the groups) of 0.6 at 80% power with a two-tailed significance level of 0.05. Using the formula above, a total sample size of 40 was obtained for each group. Owing the attrition of dropout rates of 5%, the sample size required is 80 (40 on each arm). To avoid issues of bias in participant selection, all patients will be selected from the chest clinic at the hospital. According to the chest clinic records, both sexes and different adult age groups (usually 45 years and above) are presented on each clinic day. Numbers 1 and 2 will be put on small pieces of paper, which will be placed in sealed and unmarked brown envelopes. Each participant will be asked to pick one envelop that will determine to which group she or he will belong to; this will assist in avoiding the selection bias. In addition, to ensure that the sample size is reached, discussions will be done with the head of the chest clinic to schedule more patients with CCF within the study participants' enrolment period. According to the available recommendations worldwide, 10 patients are allowed to exercise using a circuit training approach for an hour, and 3-4 therapists must be available to supervise the program to ensure safety. Because access to the Physiotherapy department at QECH to implement this pilot intervention is in the afternoon (13:00-12:00pm), and the number of patients enrolled into the active Arm is 40, the College of Medicine Physiotherapy gym will be used; and therapy session will be every Wednesday from 8:30 am to 12:30 pm. Nevertheless, the Physiotherapy department at QECH will be actively involved in the care delivery of these patients at College of Medicine Physiotherapy gym throughout the study, for capacity building and to ensure continuity of care after the implementation of this study. The participants shall be recruited over a period of two months.

4.5 Intervention and implementation procedure

Patients will be referred for CR from the medical team in the chest clinic; the referral shall be based on the inclusion and exclusion criteria. The referring of patients for screening and enrolment shall be terminated after the eight-week period. All patients will be screened again for CR eligibility using the set inclusion and exclusion criteria; following which a thorough assessment will be conducted for each patient (Appendix 10) . Briefly, the following parameters will be recorded for each patient; demographic data, medical and social history (including medications and dosages), resting Blood Pressure (BP), Pulse Rate (PR), Respiratory Rate (RR), Body Weight (BWT), Height (H), Body Mass Index (BMI) and exercise capacity.

Furthermore, risk stratification will be conducted to ascertain the risk for cardiac events during the exercise therapy; although the low-moderate risk category of patients will be offered close supervision to ensure safe exertion, all high risk patients will be excluded from exerting. In addition, each patient will be risk stratified for disease progression, to ascertain availability of risk factors and the risk for rapid progression of the disease; this will assist the therapists to plan and deliver an effective education and counselling intervention to influence life style modification. Thereafter, exercise tolerance test will be conducted using 6-minute walk test; this will assist therapists to prescribe the appropriate and safe target exertion zone at which each patient must exercise to gain positive results.

FITT (Frequency, Intensity, Time and Type) principle will be used to prescribe the exercise therapy. Table 1 describes the parameters which will be used to formulate the dosage of the exertion therapy for each patient. Using the prescription, each patient will also be advised on which physical activities they can safely do at home by themselves to promote fitness. During the therapy session, patients will be educated about the risk factors for CVD and they will be encouraged to eradicate the exposure or existence of the factors around them. Also, patients will be taught about healthy eating and importance of adhering to the medications and exercise therapy. BP and PR will be checked before and after each session to optimize safety of the patients. Also, each patient will be asked if they are facing challenges at home that are related to utilization of the therapy and exerting

at home; these will be recorded and where possible solutions will be devised. Thereafter, exercise therapy will be implemented and patients will do exactly what they were taught to do on the initial screening day and supervision will be offered throughout treatment sessions.

While participants in the Arm 1 of the trial receive both treatment modalities of CR (counselling/education and exercise therapy) and their cardiologists' prescribed cardiac medications, participants in the Arm 2 will receive the prescribed cardiac medications and education about their condition (nature of disease, risk factor modification, compliance to medications, nutritional needs); participants in Arm 2 (the control group) will come at baseline, and after 4, 8, 12 and 24 weeks for assessment and education. Figure 2 illustrates the circuit training program that will be followed during the therapy sessions. The exercise session will start with a warm up session and end with a cool down session; both sessions will be done in a group. The Arm 1 participants shall be asked to carry out two therapy sessions for each week at home, and attend a session each week at the hospital. This will assist in reducing the travel costs and avoid losses to follow up. Nevertheless, supervision at home is essential to ensure safety of the patients and efficacy of the treatment at home. Therefore, one guardian for each participant will be trained on how they will supervise the therapy sessions at home. A one-day training for the guardians will be given. All patients will be given the same home-therapy program; box stepping, walking and jogging for a predetermined FITT based on each participants' exertion capacity. The intervention will be delivered over a 12 weeks' period; participants will visit the hospital once a week and perform the rest of therapeutic tasks at home on two extra days per week for the 3 months. After the twelve-week period, all participants will be followed up for a period of twelve more weeks. All COVID-19 preventive measures namely; hand hygiene, social distance and surface/equipment disinfection will be observed. Therapists will use surgical masks during exercise therapy sessions, and patients will be asked to put on the masks before and after the exercise therapy session because they may not be able to breath while exerting.

Table 1. The reference benchmark for exercise prescription

	Frequency	Intensity	Time	Type
Cardiovascular Training (exercises to enhance cardiovascular endurance)	3 times a week for 12 weeks	40-70% Heart Rate Reserve (HRR) 12-14 Rating of Perceived Exertion (RPE)	55 minutes (15 minutes warm up, 30 minutes conditioning phase, 10 minutes cool down)	Aerobics (treadmill, board stepping, cycling, point marching)
Strength training (Exercises to improve muscular strength)	2 times a week for 12 weeks	30-70% 1 Repetition Maximum (1RM) (3040% 1RM: upper body, 50-60% 1RM: lower body)	2-4 sets of 10-15 repetitions	Resistant exercises (8-10 different muscle groups: weight lifting and pulleys, thera-band stretches)

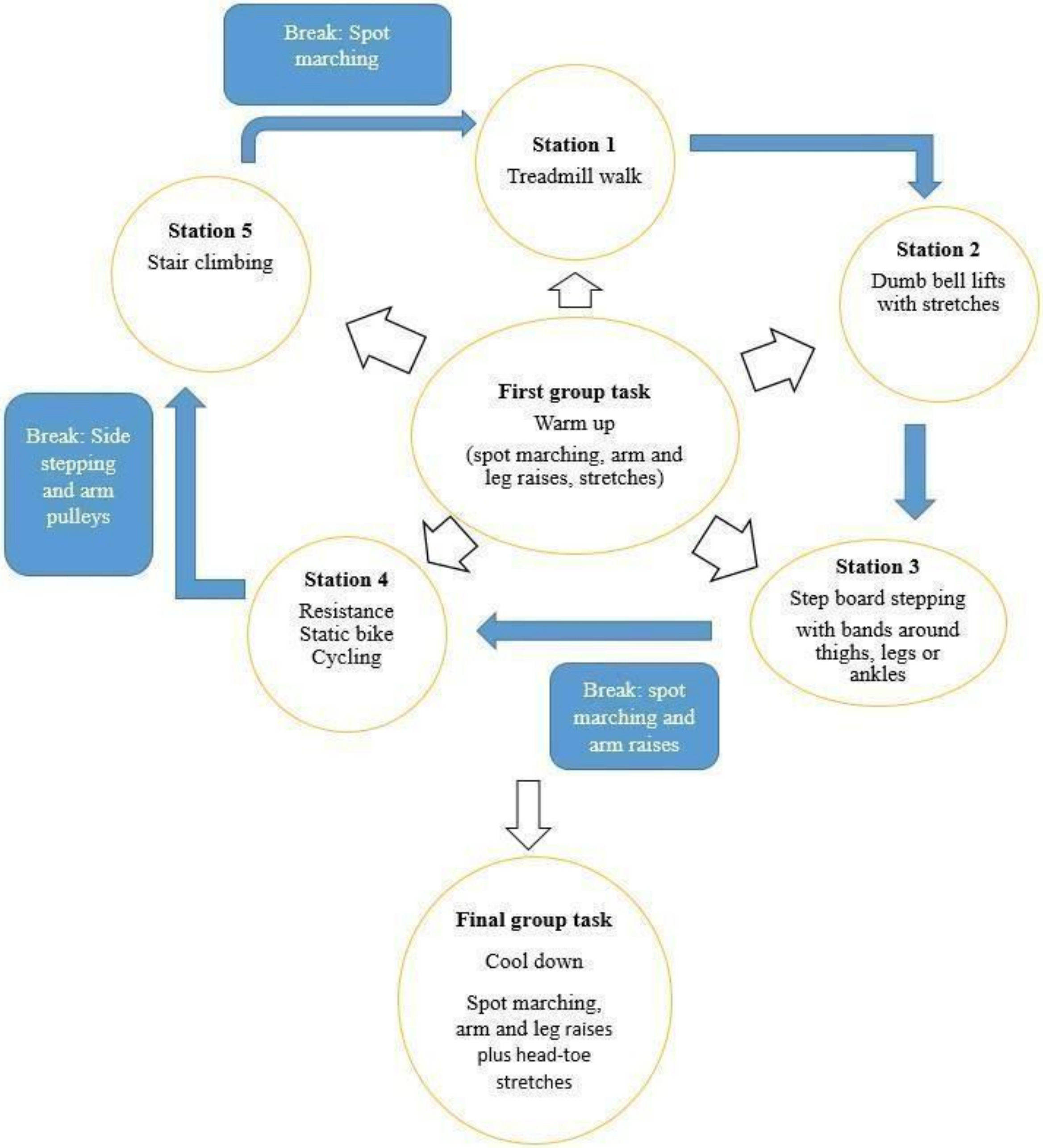

Figure 2: A circle showing the stages which will be followed during the delivery of exercise therapy, a component of CR intervention

4.6 SAFETY CONSIDERATIONS

As mentioned in section 4.5, all patients will be risk stratified for risk of cardiac events during exercise therapy. The risk stratification checklist is attached as an appendix (1) to this document. All patients classified as high risk will be excluded from participation in the study. In addition, only CR trained and registered Physiotherapists will be employed to administer the CR intervention to these patients. Furthermore, all patients will be assessed (BP, PR and general wellbeing) before and after each exercise therapy session to ensure that they are safe to exert and to exit the clinic soon after the therapy session respectively. Additionally, the study will only enroll patients who are also on secondary prevention medications; and therapists will ensure that all enrolled participants are adhering to their medications on each session day. Finally, one guardian for each patient will be trained on how they can supervise a prescribed home exercise program to ensure that patients exert safely at home. In case an adverse event such as syncope, cardiac arrest or sudden death has been recorded amongst the study population within 24 hours of exercise therapy, a proper documentation will be done and the case will be thorough investigated to ascertain if exercise therapy is indeed the cause. This and all proceedings of the investigations will also be reported to COMREC immediately in writing. In a situation where the causation is ascertained and exercise therapy is found to be at fault, the intervention will be terminated.

4.7 Data Collection

A manual sphygmomanometer will be used to measure BP, Oximeter will be used to measure Oxygen saturations. PR will be checked manually by feeling the radial artery using the third and index fingers Risk stratification will be done using the BACPR stratification forms (Appendices 1-2). Exercise testing will be conducted using Chester step test for prescription (Appendix 9), and exertion tolerance capacity will be checked using 6-minute walk test; more details on 6-minute walk test can be accessed using this link (https://doi.org/10.1164/ajrccm.166.1.at1102) (24), and outcomes of 6-minute walk test will be recorded using the form attached as an appendix (Appendix 3).

As aforementioned, patients will be screened at baseline before being enrolled into the CRP. At baseline, the following descriptive data will be collected; BP, PR, O_2sats and exercise capacity.

Additionally, data on risk stratification for disease progression and occurrence of cardiac events during exercise therapy will be recorded at baseline. BP and PR will be checked before and after each session for safety purposes (Appendix 12); patients will also be asked, on each hospital therapy day, about the challenges faced that may be negatively contributing to adherence of the home exercise and nutrition interventional advice given to be followed at home. All parameters, including incidents of hospital admissions, will also be examined and recorded at 4, 8, 12, 16 and 24 weeks. The Physiotherapists will record challenges they experienced at all stages of this project; from enrolment of study participants to discharge (Appendix 11). The data will be collected on paper, and transferred onto Microsoft excel sheet on a computer.

5.0 Data management and quality control

At the end of each therapy session, the principle investigators (PI) will check the data recording forms for data recording errors and refer any need for amendment or completion of missing data back to the research assistants. Thereafter, the PI will complete coding of all relevant fields on the data recording forms. Data will then be double entered into a secure password protected

electronic database, excel sheet, checked for errors and cleaned. The final database will be exported into Statistical Package for Social Sciences (SPSS) software for analysis. All data collecting tools, with captured information, will be stored in lockable storage boxes, and will be accessed by the PIs only.

All research assistants will be qualified Physiotherapists who are providing care to patients at QECH; such personnel have background knowledge of the tools intended to be used in this study and have a background information on CR. In addition to this, they will be trained on how to efficiently and effectively use all tools. Therefore, standard operating procedures shall be developed and distributed to all assistants to ensure that they all execute the same functions exactly in the same manner throughout the implementation of the intervention and collection of data.

6.0 Analysis and presentation of results

The SPSS software will be used to analyze this data. Frequencies and mean changes with standard deviations will be used to descriptively illustrate the effect size of the intervention. Paired t-tests will be carried out to compare and show the changes in the variables at the start of the program and after 3 and 6 months of CR reception. Independent t-tests will be carried out to compare results between the two groups. Tables and graphs will be used to present the findings.

7.0 Dissemination of results

The generated information will be presented to the multidisciplinary team involved in the care delivery to the cardiac patients at QECH. The findings will also be shared with the health care policies' key stakeholders during 2020 Malawi College of Medicine research dissemination conference. Furthermore, the results will be submitted to a peer review journal for publication.

8.0 Ethical issues

Permission will be sought from the QECH director and heads of Physiotherapy and cardiac clinics. Informed consent (Appendices 5-8) will be sought from each patient before being enrolled into the

program. All patients will be screened for safety before implementing the exercise therapy. The data will only be accessible to the therapists involved in the delivery of this intervention and the investigators. All participants' transportation costs and time will be compensated on each hospital visit and will be given $10 each

9.0 Anticipated challenges

It is anticipated that patients may be lost to follow up and discontinue with the prescribed intervention due to transportation related costs. Therefore, the patients enrolled in this program will be assisted with the top-up transportation costs; patients enrolled will be those who will manage to travel the distance with the top-up allowance. This implies that patients enrolled in the study will be those coming from within Blantyre and immediate surrounding areas. In addition, the therapy sessions have been divided into two; one will be done at the hospital and two at home.

10.0 Requirements, timeline of events and budget

Table 2: Required items for the execution of this study

ITEM	ROLE/USE
PERSONNEL: Physiotherapist<br>Cardiologist/physician<br>Statistician<br>Patients' guardian	To assess and deliver the intervention package<br>To refer patients for enrollment and respond to all relevant medical queries<br>To assist with data management and analysis<br>To guide and monitor home exercise treatment
Training: Physiotherapists & chest medical team<br><br>Patients' guardian	Briefing on the objectives of the study, the inclusion and exclusion criteria, the intervention package and their responsibilities. Testing the tools and intervention to evaluate the capacity of the health care workers to function effectively towards this study.<br>To equip them with knowledge and skills on how they can effectively guide and monitor a 2-day home exercise program every week for 12 weeks
Paper: 3 realms	To print all checklists and reference guides, study information sheets, consent forms and to aide in the data management and report writing
Transport: 1 or 2 tone	To assist in transporting the equipment and all other accessories from the shop to the clinic site
Space: clinical gym area	This will be in physiotherapy department at the hospital. The communication has been made already. In case emergency situations have occurred and the space is not conducive for our participants, we shall use COM physiotherapy clinical gym. The communication has also been made already and we have been assured of the space.

Participants' screening and assessment kit	A number of equipment and accessories (indicated in the budget) will be needed during the screening and assessment processes. These are items such as cones, timer, leaflets etc.
Exercise therapy kit	A list of equipment will also be needed for the delivery of the intervention.<br>These are items such as treadmill, stepping boxes etc.

Table 3: Timeline of events

ACTIVITY	COMPLETION DATE	RESPONSIBLE PERSON	Key authority
Ethical approval	September-October, 2020	Investigators	Investigators
Site permission	September-November, 2020	Investigators	Investigators
Training and final discussions with all stakeholders	December, 2020	Investigators	Investigators
Screening for enrollment	January-February, 2021	Investigators	Investigators
Therapy implementation	February-March, 2021	Investigator and therapists	Investigators
Data management and analysis	April, 2021	Investigators	Investigators
Report writing	May-July 2021	Investigators	Investigators
Dissemination of results	August-October, 2021	Investigators	Investigators

Table 4: Budget items for the study (1USD: 750MWK)

ITEMS	SPECIFICATION	QUANTITY	COST PER ITEM	TOTAL COST (MK)	TOTAL COST ($)	JUSTIFICATION
PERSONNEL	Cardiologist/Physician	1	MK20,000/session for 12 sessions	MK240,000	$320	For medical enquiries, such as to do with medications. This will also be a focal person in all referrals into the study.
	Physiotherapists	4	MK20,000/individual/session for 12 sessions	MK960,000	$1280	For enrollment, screening and implementation.
		2	MK20,000/individual/session for 2 extra patient visits	MK40,000	$53.3	Two therapists will be needed to conduct assessments on all patients during the follow up period at 16 and 24 weeks after the implementation period
	Statistician	1	MK20,000/day for 10 days	MK200,000	$266.67	To assist with data management

EQUIPMENT	sphygmomanometer	3	MK30,000	MK90,000	$120	
	Stethoscope	3	MK45,000	MK135,000	$180	
	Dumbbells	18 pairs	MK12,000 a pair	MK 96,000	$128	
	Stepping boxes	31	MK 17,000 a box	MK 527,000	$702.67	15 boxes will be given to the patients for home exercise program and 16 will be used at the hospital
	Stair board	-	Available			
	Thera-bands	3 (colored bundles: y, r, b)	MK50,000 per bundle	MK150,000	$200	For strength training
	Treadmill	1	MK800,000	MK800,000	$1066.67	
	Static bike	1	Available	-	-	
	Computer	1	Available	-	-	
	Kettle	1	MK20,000	MK20,000	$26.67	To prepare tea for patients and therapists

SUPPLIES	Disposable cups<br><br>Paper<br>Tea<br><br>Milk<br><br>Sugar<br><br>Hand soap<br><br>Sanitizer<br><br>Disinfectant<br><br>Surgical face masks<br><br>Toilet paper	40<br><br>3 Realms<br><br>2 packets<br><br>3 tins<br><br>5 packets<br><br>1 Litre<br><br>3 Litres<br><br>4 Litres<br><br>4 boxes<br><br>5 packets of 9 rolls	MK2500/set of 20<br><br>MK15,000 each<br>MK2000 each<br><br>MK4000 each<br><br>MK1000 each<br><br>MK 3500<br><br>MK 10,000<br><br>MK 20,000<br><br>MK 32,000<br><br>MK 10,000 each	MK5,000<br><br>MK45,000<br><br>MK4,000<br><br>MK12,000<br><br>MK5,000<br><br>MK3500<br><br>MK10,000<br><br>MK20,000<br><br>MK32,000<br><br>MK50,000	$6.67<br><br>$60<br><br>$5.33<br><br>$16<br><br>$6.67<br><br>$4.67<br><br>$13.33<br><br>$26.67<br><br>$42.67<br><br>$66.67	Needed for one session. Patients to bring own bottles for refill on all subsequent visits<br><br>The listed items shall be needed as they are necessary for patients' hydration, hygiene and sanitation. In addition, the paper will be required for data collection<br><br>Personal protective equipment for both patients and health care workers.

TRANSPORT	Vehicle<br><br>Fuel	Available<br><br>240 Litres	-<br>MK898/litre	-<br>MK215,520	-<br>$287.36	Investigator to provide To buy and collect the necessities for the study and to run all other related errands.
COMPENSATION FOR PARTICIPANTS	Transportation cost and refreshments	760 hospital visits	MK7500 per visit	MK5,700,000	$7600	15 Hospital visits fo each patient in arm 1 (40 participants), and 4 hospital visits for eacl patient in arm 2 (40 participants). Therefore,
CONTINGENCY			MK200,000	MK200,000	$266.67	
10% COM OVERHEAD FEES				MK956,002	$1274.67	
**TOTAL BUDGET COST**				MK10,516,022	$14021.4	

This pilot study is being sponsored by NCT-BRITE.

REFERENCES

1. World Health Organisation. (2017). Cardiovascular diseases. Retrieved from http://www.who.int/news-room/fact-sheets/detail/cardiovascular-diseases-(cvds)

2. Stewart, J., Manmathan, G., & Wilkinson, P. (2017). Primary prevention of cardiovascular disease: A review of contemporary guidance and literature. Journal of the Royal Society of Medicine Cardiovascular, 6, 1-9. https://doi.org/10.1177/2048004016687211

3. World Health Organization. (2014). Non-communicable Diseases: Country Profiles. Retrieved from https://www.who.int/nmh/countries/2014/mwi_en.pdf

4. Onen, C., L. (2013). Epidemiology of ischaemic heart disease in sub-Saharan Africa. *Cardiovasc J Afr, 24*(2), 34–42. https://doi/org/10.5830/CVJA-2012-071.

5. Roth, G. A., Huffman, M. D., Moran, A. E., Feigin, v., Mensah, G. A., Naghavi, M., Murray, C. J. L. (2015). Global and regional patterns in cardiovascular mortality from 1990 to 2013. *Circulation, 2015(*132):1667-1678. https://doi.org/10.1161/CIRCULATIONAHA.114.008720.

6. Brown KG, Willis WH. Cardiac disease in Malawi. S Afr Med J. 1975 May 31;49(23):926–30.

7. Murray A. [An Insight into structural heart disease in medical patients, Queen Elizabeth

   Central Hospital. A four-week elective project report submitted in partial fulfilment of the Bachelor of Medicine and Bachelor of Surgery degree at the University of

Version 2.0-03/02/21

Aberdeen]. Unpublished report. University of Aberdeen, Aberdeen, United Kingdom; 2012.

8. Allain TJ. [Summary of a logbook of echocardiograms carried out on medical patients at Queen Elizabeth Central Hospital by TJA, 2008-2013]. Unpublished raw data. College of Medicine, University of Malawi, Blantyre, Malawi; 2013.
9. Soliman EZ, Juma H. Cardiac Disease Patterns in Northern Malawi: Epidemiologic Transition Perspective. J Epidemiol. 2008;18(5):204–8.
10. Stewart S, Carrington M, Pretorius S, Methusi P, Sliwa K, Abegunde D, et al. Standing at the crossroads between new and historically prevalent heart disease: effects of migration and socio-economic factors in the Heart of Soweto cohort study. Eur Heart J. 2011 Feb;32(4):492–9
11. Ojji D, Stewart S, Ajayi S, Manmak M, Sliwa K. A predominance of hypertensive heart failure in the Abuja Heart Study cohort of urban Nigerians: a prospective clinical registry of 1515 de novo cases. Eur J Heart Fail. 2013 Aug;15(8):835–42.
12. Chillo P, Bakari M, Lwakatare J. Echocardiographic diagnoses in HIV-infected patients presenting with cardiac symptoms at Muhimbili National Hospital in Dar es Salaam, Tanzania. Cardiovasc J Afr. 2012 Mar;23(2):90–7.
13. Schwartz T, Magdi G, Steen TW, Sjaastad I. HIV as a risk factor for cardiac disease in Botswana: a cross-sectional study. Int Health. 2012 Mar;4(1):30–7.

14. Hakim JG, Matenga JA, Siziya S. Myocardial dysfunction in human immunodeficiency virus infection: an echocardiographic study of 157 patients in hospital in Zimbabwe. Heart. 1996 Aug;76(2):161–5.

15. Hakim JG, Manyemba J. Cardiac disease distribution among patients referred for echocardiography in Harare, Zimbabwe. Cent Afr J Med. 1998 Jun;44(6):140–4.
16. World Health Organisation. (1993). Rehabilitation after cardiovascular diseases, with special emphasis on developing countries. Retrieved from https://apps.who.int/iris/bitstream/handle/10665/38455/WHO_TRS_831.pdf?sequence=1&isAllowed=y
17. British Association for Cardiovascular Prevention and Rehabilitation. (2017). The BACPR standards and core components for cardiovascular disease prevention and rehabilitation. Retrieved from http://www.bacpr.com/resources/AC6_BACPRStandards&CoreComponents2017.pdf
18. Grace, S. L., Turk-Adawi, K. I., Contractor, A., Atrey, A., Campbell, N., Derman, W., … Sarrafzadegan, N. (2016). Cardiac rehabilitation delivery model for lowresource settings. Heart (British Cardiac Society), 102(18), 1449–1455. https://doi.org/10.1136/heartjnl-2015-309209
19. Association of Chartered Physiotherapists in Cardiac Rehabilitation. (2015). Standards for physical activity and exercise in the cardiovascular population (ACPICR standards, 3rd edition). Retrieved from https://www.acpicr.com/data/Page_Downloads/ACPICRStandards.pdf

20. Dalal, H., M., Doherty, P., & Taylor, R., S. (2015). Cardiac rehabilitation; A clinical review. *British Medical Journal, 351*(h5000), 1-8. https://doi.org/10.1136/bmj.h5000

Version 2.0-03/02/21

21. Anderson, L., & Taylor, R. S. (2014). Cardiac rehabilitation for people with heart disease: An overview of Cochrane systematic reviews. Cochrane Database of Systematic Reviews, 2014(12), 1-23.

https://doi.org/10.1002/14651858.CD011273.pub2.

22. Long, L., Anderson, L., Dewhirst, A. M., He, J., Bridges, C., Gandhi, M., & Taylor, R. S. (2018). Exercise-based cardiac rehabilitation for adults with stable angina. *Cochrane Database of Systematic Reviews, 2018*(2), 1-53. https://doi.org/10.1002/14651858.CD012786.pub2

23. Choo, C. C., Chew, P. K. H., Lai, S. M., Soo, S. C., Ho, C. S., Ho, R. C., & Wong, R.C. (2018). Effect of cardiac rehabilitation on quality of life, depression and anxiety in Asian patients. International Journal of Environmental Research and Public Health, 15(6), 1095. https://doi.org/10.3390/ijerph15061095

24. American Thoraccic Society. (2002). ATS statement guidelines for 6-minute walk test. American Journal of Respiratory and Critical Care Medicine, 166(1):1-7. https://doi.org/10.1164/ajrccm.166.1.at1102

25. Awotidebe, T., Adedoyin, R., Balogun, M., Adebayo, R., Adeyeye, V., I. Oke, K., Ativie, R., Akintomide, A. and Akindele, M. (2016). Effects of Cardiac Rehabilitation Exercise Protocols on Physical Function in Patients with Chronic

Heart Failure: An experience from a resource constraint nation. *International Journal of Clinical Medicine*, **7**, 547-557. https://doi.org/10.4236/ijcm.2016.78060.

APPENDICES

Appendix 1: Risk stratification for cardiac events

LOW-RISK	MODERATE RISK	HIGH RISK
• Left ventricular ejection fraction > 50%<br>• No resting or exerciseinduced complex dysrhythmias<br>• Uncomplicated MI, CABG, angioplasty, atherectomy, or stent:<br>• Absence of CHF or signs/symptoms indicating post-event ischemia<br>• Normal hemodynamic and ECG responses with exercise and in recovery<br>• Asymptomatic with exercise or in recovery, including absence of angina<br>• Maximal functional capacity at least 7.0 METs*<br>• Absence of clinical depression or depressive symptoms	• Patients neither meet High Risk nor Low Risk standards;<br>• Left ventricular ejection fraction = 40–50%<br>• Signs/symptoms including angina at “moderate” levels of exercise (60–75% of maximal functional capacity) or in recovery<br>• Mild to moderate silent ischemia (ST depression less than 2mm) with exercise or in recovery	• Left ventricular ejection fraction < 40%<br>• Survivor of cardiac arrest or sudden death<br>• Complex ventricular dysrhythmias (ventricular tachycardia, frequent [> 6/min] multiform PVCs) at rest or with exercise<br>• MI or cardiac surgery complicated by cardiogenic shock, CHF, and/or signs/symptoms of post-procedure ischemia<br>• Abnormal hemodynamics with exercise, especially flat or decreasing systolic blood pressure or chronotropic incompetence with increasing workload<br>• Significant silent ischemia (ST depression 2mm or greater without symptoms) with exercise or in recovery<br>• Signs/symptoms including angina pectoris, dizziness, lightheadedness or dyspnea at low levels of exercise (< 5.0 METs) or in recovery<br>• Maximal functional capacity less than 5.0 METs*

		• Clinically significant depression or depressive symptoms

Appendix 2: Risk stratification for cardiovascular disease progression

**Positive Risk Factors**	**Defining Criteria**	**Points**
Age	Men ≥ 45 years, Women ≥ 55 years	+1
Family History	Myocardial infarction, coronary revascularization, or sudden death before 55 years of age in father of other 1st degree male relative or before 65 years of age in mother or other 1st degree female relative	+1
Cigarette Smoking	Current cigarette smoker or those who quit within the previous six months, or exposure to environmental tobacco smoke (i.e., secondhand smoke)	+1
Sedentary Lifestyle	Not participating in at least 30 minutes of moderate-intensity physical activity on at least three days/week for at least three months	+1
Obesity	Body mass index ≥30 kg/m2 or waist girth >102 cm (40 inches) for men >88 cm (35 inches) for men	+1
Dyslipidemia	Low-density lipoprotein (LDL) cholesterol ≥ 130mg/dL (3.37 mmol/L) or high-density lipoprotein (HDL) cholesterol <40mg/dL (1.04mmol/L) or currently on lipid-lowering medication; If total serum cholesterol is all that is available, use serum cholesterol >200 mg/dL (5.18mmol/L)	+1
Prediabetes	Fasting plasma glucose ≥100 mg/dL (5.50 mmmol/L) but <126 mg/dL (6.93 mmol/L) or impaired glucose tolerance (IGT) where a two-hour oral glucose tolerance test (OGTT) value is ≥140 mg/dL (7.70 mmol/L), but <200 mg/dL (11.00mmol/L)	+1
**Negative Risk Factors**	**Defining Criteria**	**Points**
High HDL Cholesterol	≥60 mg/dL (1.55 mmol/L)	-1

TOTAL CVD SCORE	

Appendix 3: 6-Minute Walk test recording form

Patient ID:

- ○ Medical history checked ○ Medical clearance provided for the patient to participate in exercise testing **Contraindications to 6MWT:**
- ○ Resting heart rate > 120 beats / min after 10 minutes rest (relative contraindication) ○ Systolic blood pressure > 180 mm Hg +/- diastolic blood pressure > 100 mm Hg (relative contraindication) ○ Resting SpO2 < 85% on room air or on prescribed level of supplemental oxygen ○ Physical disability preventing safe performance ○ No contraindications identified

**6MWK**						**Date:**	**Time:**
**Supplemental oxygen?**						**Mobility aid?**	
**Time (mins)**	**BP**	**SPO_2**	**HR**	**RPE**	**Distance walked**	**Rests/Comments**	
**Rest**							
**1**							
**2**							
**3**							
**4**							
**5**							
**6**							
**Recovery 1**							
**2**							

**Total distance:**	**Symptom recovery:**	**Heart rate recovery:**
**Limiting factor:**		
**Was test terminated? Yes No**	**If yes when?:**	

Appendix 4: Cardio-respiratory parameters

ID: **Initials:**

PARAMETERS	BASELINE	4 WEEKS	8 WEEKS	12 WEEKS	16 WEEKS	24 WEEKS
Blood Pressure						
Heart Rate						
O_2sats						
Exercise tolerance capacity						
Challenges						
Number of hospital admissions (reason for admission)						

Appendix 5: English version letter with the detailed information regarding the study for consenting purposes

University of Malawi, College of Medicine-Physiotherapy Department
Detailed information regarding your participation in a research study:

Impact of cardiac rehabilitation on cardiorespiratory parameters: Towards establishment of cardiac rehabilitation intervention at Queen Elizabeth Central hospital

Dear participant,

Study Purpose

You are being asked to participate in a research study being conducted by lecturer in Physiotherapy Department at the University of Malawi, College of Medicine. The purpose of this study is to examine the impact of cardiac rehabilitation on cardiorespiratory parameters at queen Elizabeth central hospital.

Study Procedures

If you decide to participate in this study, you will be asked to undergo a 12-week cardiac rehabilitation program. This is an intervention designed for patients suffering from a cardiovascular disease. The intervention package constitutes exercises, counselling/education aimed at minimising and eliminating existence of risk factors, and monitoring the adherence to the secondary prevention medications. You will be screened on entry to this study, and a brief screening will be repeated each day you come to the hospital for this treatment. You will be asked to come to the hospital for this

treatment once a week or after 8 and 12 weeks from the day you enrol into the study. Within each week, you will be expected to exercise at home twice or engage in performing physical activities. Upon completion of the 12 weeks, you will be asked to report to the hospital on the 16th and 24th week to have yourself screened again. The screening findings and all information obtained from you will be kept strictly confidential, and your name will not be associated with the information that appears in our report. You shall be assigned an identity code as opposed to your name, and the codes will be used during analysis of our findings.

Possible Risks

During exercise therapy, severely affected individuals may experience exercise induced ischemia. These are patients who have been classified as high risk for cardiac events on risk stratification for cardiac events during exertion. Therefore, all patients under this category have been excluded from participation. Conclusively, it is safe for you to exercise if you have been screened and you are classified as low or medium risk patient. Also, social distance, hand washing and disinfection of surfaces and therapy equipment will be observed to avoid COVID-19 transmissions.

Possible Benefits

It is expected that the outcomes of this study will assist us to develop a cardiac rehabilitation intervention that is effective and respond to the health needs of cardiac patients at this hospital. As a patient, we anticipate that this intervention will help to improve your health by positively affecting your cardiorespiratory parameters such as blood pressure, pulse rate, and exercise capacity. The findings of the study will also help us inform policy makers, health care practitioners and other stakeholders to consider incorporation of this intervention in the management protocol for cardiovascular disease in Malawi. It is also anticipated that information gained from this study will help us to answer some of the important questions about the efficacy of cardiac rehabilitation in resource constrained areas such as Malawi.

Participation compensation

Your transportation costs will be compensated for; you will receive MK7500.00 on each hospital visit day for this.

Voluntary Participation

Participation in this study is completely voluntary. You are free to refuse participation in this study. Your decision regarding participation in this study will not affect you in any way. If you decide to participate, you are free to change your mind and discontinue participation at any time.

Confidentiality

Information about you obtained for this study will be kept confidential. Your name and other identifying information will not be kept with the interview information. It and this consent form will be kept in separate, locked file cabinets, and there will be no link between the consent form, the questionnaire and the interview. The information obtained will not be made available to anyone else. Any reports or publications about the study will not identify you or any other study participant.

Questions

Any study-related questions, problems or emergencies should be directed to the following researcher:

Alice Namanja 0997759408

Questions about your rights as a study participant, comments or complaints about the study also may be presented to the College of Medicine, Research and Ethics Committee, private bag 360, BT 3, or by telephone to 011 871 911 or by extension line on 334.

Appendix 6: English version of the consent form

University of Malawi, College of Medicine-Physiotherapy Department Consent to participate in a research study:

Impact of cardiac rehabilitation on cardiorespiratory parameters: Towards establishment of cardiac rehabilitation intervention at Queen Elizabeth Central hospital

I have read the information provided in the participant's information sheet, and am satisfied with my understanding of the study, its possible benefits, risks and alternatives. My questions about the study have been answered. I hereby voluntarily consent to participation in the research study as described. I have been offered copies of this two-page information sheet and consent form. * * *

______________________________	______________________________
Signature of participant	Date
______________________________	______________________________
Thumbprint	Date
______________________________	______________________________
Name of participant (printed)	Witness

Appendix 7: Chichewa version letter with the detailed information regarding the study for consenting purposes

University of Malawi, College of Medicine-Physiotherapy Department
Detailed information regarding your participation in a research study:

Impact of cardiac rehabilitation on cardiorespiratory parameters: Towards establishment of cardiac rehabilitation intervention at Queen Elizabeth Central hospital

Wokondedwa Mayi/Bambo

Cholinga cha kafukufuku

Kafukufukuyu akupangidwa ndi mphunzitsi wa sukulu yaukachenjede ya University of Malawi, College of Medicine, mu department ya Physiotherapy. Cholinga chakafukufukuyu ndikufuna kudziwa za momwe thandizo lomwe lakonzedwa kuti lipelekedwe kwa anthu omwe alindimatenda a mtima, pachipatala cha Queen Elizabeth Central hospital, lingawathandizire posintha mthupi mwawo.Thandizoli likutchedwa cardiac rehabilitation mu chingerezi.

Ndondomeko ya kafukufuku

Mukavomeleza kutenga gawo mu kafukufukuyu, mupemphedwa kulandira thandizoli kwa masabata khumi ndi awiri (12). Thandizoli lili ndi zinthu ngati izi; mafizo, uphungu wazaumoyo omwe uzipelekedwa ndicholinga chothandizira kuchepetsa kapena kuchotseratu zinthu zomwe zimanyanyisa matenda a mtima mthupi lamunthu, komanso chilimbikitso chopelekedwa kwa odwalawa kuti azimwa mankhwala oteteza mtima omwe apatsidwa ndi adokotala awo mwandondomeko. Thandizoli likuunikidwa kuti tione ngati lingathandizire kusintha mwa ubwino kuthamanga magazi, phuma komanso kumuthandiza wodwala kuti akhale wotha kuziimira payekha

pakutakataka ntchito zapakhomo or kugwira ntchito zina ndi zina ndi mphamvu. Mudzapimidwa musanayambe kulandira thandizoli, komanso nthawi iliyonse yomwe mukubwera kudzalandira thandizoli mudzapimidwa. Kupimaku kudzathandizira kuti mulandire thandizo lokuyenerezani. Mudzapemphedwa kubwera kuchipatala tsiku limodzi pasabata kapena pakatha masabata 8 ndi 12, kuchokera tsiku loyamba lomwe mwalowera mukafukufukuyu kudzalandira thandizoli, mpaka masabata onsewa athe. Pa sabata iliyonse, mudzapemphedwa kuchita mafizo masiku awiri pakhomo pano kapena kukhala otakataka pakugwira ntchito zapakhomo panu. Masabata khumi ndi awiri akatha, mudzapemphedwa kufika kuchipatala pakapita mwezi umodzi ndi miyezi itatu kuti mudzapimidweso.

Zotsatira za kupimidwa kwanu komanso uthenga onse otengedwa uzasungidwa mwa chinsisi. Uthengawu sudzaonesa mayina anu. Mudzapatsidwa nambala yachinsinsi yomwe idzagwiritsidwe ntchito pounikira uthenga wanu ndi wa anthu ena.

Chiopsyezo chotsatira mkafukufukuyu

Odwala omwe apezeka kuti alipachiopsyezo chomva ululu pamtima kwambili chifukwa cha mafizo sakuyenera kutenga gawo mukafukufukuyu. Kotero, onse omwe alibe chiopsyezochi ndiomwe akuloledwa kutenga gawo mu kafukufukuyu. Kukhala motalikana, kusamba mmanja komanso kupukuta ziwiya zomwe zikugwiritsidwa ntchito popeleka zipangizozi zizatsatidwa pofuna kupewa kufala kwa nthenda ya COVID-19.

Zopindula mkafukufukuyu

Kafukufukuyu athandizira kuunikira mmene thandizoli lingathandizire anthu omwe akuvutika ndinthenda ya mtima. Zotsatira za kafukufukuyu zithandiziranso kuti thandizoli likonzedwe bwino moyenelera ndizosowa za odwalawa. Inu ngati odwala, kafukufukuyu akuthandizaniso kupeza bwino mthupi mwanu. Komanso, kafukufukuyu athandizira kuti onse okhuzidwa popanga malamulo ndi ndondomeko zoyendetsera zaumoyo akonze ndondomeko zoyenelera mdziko lathu

lino, kuti anthu omwe ali ndi matendawa alandire thandizo loyenelera. Kafukufukuyu athandizanso kuti tidziwe mmene thandizo limeneli lingathandizilile anthu odwala matenda amenewa mmadera osauka ngati mdziko lathu lino la Malawi.

Kangachepe kolandira Kamba ka nthawi yanu

Nthawi iliyonse yomwe mukubwera kudzalandira thandizoli mudzapatsidwa ndalama yokwana MK7500.00, yomwe mukuyenera kugriritsa ntchito pamayendewe anu kuchoka ndikubwelera kunyumba kwanu. Madzi akumwa komanso tiyi adzapelekedwa tsiku lililonse lomwe mudzabwera kuchipatala kudzalandira thandizoli.

Ufulu wakutenga nawo gawo

Muli ndi ufulu otenga gawo mukafukufukuyu kapena kukana kutero. Chisankho chanu chakuvomela kapena kukana kutenga gawo mukafukufukuyu sikubweletsa vuto lililonse kwa inu. Mukasankha kutenga nawo gawo mu kafukufukuyu, dziwani kuti muli ndi ufulu osiyira panjira ngati mwasintha maganizo.

Chinsinsi

Mapepala onse omwe agwiritsidwa ntchito mu kafukufukuyu, potolela uthenga kuchokera kwa inu, ndimthupi mwanu sizizaikidwa malo oonekela, ndipo zizaonedwa ndi yekhayo yemwe akupanga kafukufukuyu zikasungidwa. Uthenga otumizidwa kumabungwe, mzipatala ndi anthu ena okhuzidwa, suzakhala ndi mayina anu.

Mafunso

Ngati muli ndi mafunso kapena chidandaulo chokhudza ndi kafukufukuyu, kapena kayendetsedwe ka kafukufukuyu, yimbani lamya kwa;

Alice Namanja 0997759408

Ngati muli ndi mafunso okhuza ndi ufulu wanu kapena chidandaulo chokhudzana ndi kafukufukuyu mukhozanso kufikila a College of Medicine, Research and Ethics Committee, private bag 360, BT 3, kapena imbani lamya pa 011 871 911 or by extension line on 334.

Appendix 8: Chichewa version of the consent form

University of Malawi, College of Medicine-Physiotherapy Department Consent to participate in a research study:

Impact of cardiac rehabilitation on cardiorespiratory parameters: Towards establishment of cardiac rehabilitation intervention at Queen Elizabeth Central hospital

Ndawelenga ndipo ndakhutitsidwa ndizonse zomwe zalembedwa mu chikalata chokhuza ndi kafukufukuyu. Mafunso anga onse ndi madandaulo anga onse ayankhidwa bwino. Ndikuvomeleza mwakufuna kwanga kutenga nawo gawo mukafukufukuyu. Uthengawu ndapatsidwa kuti ndizikumbukira zofunikira ndi zonse zokhuzana ndikafukufukuyu.

* * *

________________________________ ________________________________ Signature ya otenga mbali Date

________________________________ ________________________________

Chidindo cha chara Date

________________________________ ________________________________

Dzina la otenga mbali (printed) Mboni

Appendix 9: Chester step test, 1RM test findings recording and exercise prescription form

PATIENT ID:………

HR AT REST………

BETA-BLOCKED?:……

HRMAX:………

HRR:………

70%HRR:……

STAGES	HEART RATE	METS ACHIEVED	OXYGEN SATURATION	RPE
1				
2				
3				
4				
5				

TEST COMPLETED? YES NO

REASON FOR RMINATION:………………………………………………………………………………………………

……

……

……

1 REPETITION MAXIMUM

1RM:

PRESCRIPTION (40-60% HRRMAX)

Results in use: HR……… METS achieved:………. RPE:

Prescribed zone: %HRR………. METS max: RPE:

CV ENDURANCE

TREADMILL	CYCLING	STAIR STEPPING	PHYSICAL ACTIVITIES

STRENGTH TRAINING ULS

(30-40% 1RM):

LLS (50-60% 1RM):

HOME PROGRAM

EXERCISE	DOSAGE	PHYSICAL ACTIVITIES ENCOURAGED?
STAIR STEPPING		
STRENGTH TRAINING		
WALKING		

Appendix 10: Patient Assessment form

PATIENT ID: RACE:

AGE: LOCATION:

GENDER:

CHIEF COMPLAINT:
………
………
………
………
………
………
………
………
………
……

HISTORY OF PRESENTING COMPLAINT:
………
………
………
………
………
……

CORMOBIDITIES:
………

………
……

INVESTIGATIONS DONE:
………
………
………
………
………
………
………

MEDICATIONS:
………
………
………
………
……

SOCIAL HISTORY:
………
………
………
………
………
……

FUNCTIONAL ABILITIES:
………
………
………
………
………
………
………
………

VITALS: ………..

WEIGHT: HEIGHT: BMI:

MUSCULOSKELETAL (ROM+POWER):
………
………
………
………
………
………
………

CHEST ASSESSMENT:
………
………
………
………
………
………
………
……

FUNCTIONAL ASSESSMENT TESTS (6-MINUTE WALK TEST/CHESTER STEP TEST): If done, record on the forms provided.

Appendix 11: Checklist for reporting challenges encountered by the Physiotherapists

Physiotherapist ID:

	MULTIDISCIPLINARY/INTER DISCIPLINARY TEAM WORK	PATIENT ENGAGEMENT	RESOURCE MANAGE ENT	EXPERTISE
ENROLLMENT				
BASELINE ASSESSMENT				
8 WEEKS				
12 WEEKS				
16 WEEKS				
24 WEEKS				

Appendix 12: Weekly vital signs recording form

Patient ID:

SESSI ƆN	DATE	BP		HR		OXYGEN SATURATION	
		BEFORE ET	AFTER ET	BEFORE ET	AFTER ET	BEFORE ET	AFTER ET
1							
2							
3							
4							
5							
6							
7							
8							

9							
[illegible]0							
[illegible]1							
[illegible]2							

COLLEGE OF MEDICINE

Principal

M. H. C. Mipando, MSc, PhD.

College of Medicine
Private Bag 360
Chichiri
Blantyre 3
Malawi

Our Ref.:

Telephone: 01 871911
01 874107

Your Ref.:

Fax: 01 874 700

28th September,2020

The Chairman
College of Medicine Research and Ethics Committee (COMREC)
P/ Bag 360
Chichiri
Blantyre 3
RE: LETTER OF SUPPORT – Ms. ALICE NAMANJA

The subject above refers;

Alice Namanja is a lecturer in the department of Physiotherapy, College of Medicine. Alice secured funding from NCD-BRITE to conduct a study titled "Impact of Cardiac Rehabilitation on cardiorespiratory parameters: Towards establishment of CR intervention at Queen Elizabeth Central Hospital". The Physiotherapy department is aware of this work
and I will support her.

Please assist her accordingly.

E. Chisati
Head of Physiotherapy Department

Telephone: (265)874 333/877 333
Facsimile: (265)874 603
Email: queenshospital@malawi.net

All *Communications should be addressed to:*
The Hospital Director

In reply please quote No. ……………..……
QUEEN ELIZABETH CENTRAL HOSPITAL
MINISTRY OF HEALTH
P. O. BOX 95
BLANTYRE
MALAWI

19th January 2021

The Chairperson
College of Medicine Research and Ethics Committee
College of Medicine
Private Bag 360
Chichiri
Blantyre 3

Dear Sir,

Impact of Cardiac Rehabilitation on cardiorespiratory parameters: A pilot study towards establishment of CR intervention at Queen Elizabeth Central Hospital

I write to support the conduct of the above proposed research to be done in this hospital by Ms Alice Namanja. We are having an increased number of patients with cardiac patients and we believe this intervention will optimise their care.

We request that the findings of the study be made available to our Hospital Research Committee and also the relevant departments in this hospital.

We wish the study team success.

Yours faithfully

The Hospital Director
Queen Elizabeth Central Hospital

Dr K. Mponda
ACTING DEPUTY HOSPITAL DIRECTOR - CLINICAL

MEDICAL COUNCIL OF MALAWI
P O BOX 30787, LILONGWE 3

ANNUAL REGISTRATION RENEWAL CERTIFICATE

I HEREBY CERTIFY that the person whose name and original Registration number appear herein, having duly complied with the provisions of the Medical Practitioners and Dentists Act, 1987 and the Regulations made thereunder respecting registration, is entitled to practice as

PRACTICE : **Physiotherapist**

NAME : **Alice D.J. Namanja**

REG. NO. : **MCM/PHY/0143**

This certificate is evidence of registration until **30 June 2021**

THIS CERTIFICATE IS NOT A PRIVATE PRACTICE LICENCE

IMPORTANT – **Please read the notes overleaf**

CURRICULUM VITAE

Alice Namanja
University of Malawi
College of Medicine
Physiotherapy Department
Cell: 0997759408/+447919965574
Email: anamanja@medcol.mw/ alicenamanja6@gmail.com

---

1. PERSONAL INFORMATION

Name: Alice Namanja
Nationality: Malawian
Gender: Female
Marital Status: Single
Date of birth: 18th August 1991

PROFILE STATEMENT

I am a logical and strategic thinker, with appropriate and good communication skills. I am friendly and a hard worker, and I am currently working towards championing cardiac rehabilitation in a developing country. In the next 5-10 years, I would like to be a competent academician and physiotherapist working within a specialty of cardiac and respiratory rehabilitation.

2. EDUCATION QUALIFICATIONS

University of Chester (2018-2019)

MSc Cardiovascular Health and Rehabilitation (ongoing)

Modules included cardiovascular diseases, risk factors, investigations and treatments, cardiovascular anatomy and exercise physiology, clinical exercise testing and prescription, nutrition and cardiovascular health, research methods and data analysis and physical activity and exercise programming in the management of cardiovascular disease. My dissertation project is investigating the impact of cardiac rehabilitation treatment modalities in SubSaharan Africa.

University of Malawi, College of Medicine (2010-2014)

Bachelor of Physiotherapy with Second Upper class

Modules included A-level subjects (biology, chemistry, computer skills, English language and communication, mathematics and physics), professional orientation, structure and organisation of the human body, human physiology, biochemistry, microbiology, Movement science, introduction to general pathology, body movement and function, basic sciences applied to physiotherapy, child growth and development, applied physiotherapy, physiotherapy treatment and intervention, clinical physiotherapy, physiotherapy in the community, research methods and management and administration. My final year project examined the awareness of the intensive care nurses on the role of physiotherapy in the management of critically ill patients.

Providence Girls Secondary School (2005-2008)

Malawi school certificate of education with 13 points

O-level subjects that included physical science, biology, mathematics, agriculture, social and development studies, computer studies, English language, Chichewa language and French.

3. CONTINUING PROFESSIONAL DEVELOPMENT

- Certificate in education (Online-based); Obtained from University of Malawi, College of Medicine in 2018. This was obtained after undergoing 8 months online classes.
- Certificate of attendance; Teach the Teacher workshop, organized by College of Medicine Education and Training Office (COMETO) together with the University of Dundee in 2017. The facilitators were educators from Dundee, and it was a 7 days long training.
- Certificate of completion; Foundation Spirometry training. This course was delivered by the Spirometry Training Services Africa CC, in collaboration with Pan African Thoracic Society-Methods in Epidemiologic Clinical and Operations Research (PATS MECOR), July 2016. This was a two weeks class-based and laboratory course work.
- Certificate of achievement; Designing a clinical research- methods. This course was prepared and delivered by the Pan African Thoracic Society-Methods in Epidemiologic Clinical and Operations Research (PATS MECOR) July 2016. This was delivered within a period of 7 days.

- Certificate in Basic Good Clinical Practice obtained at University of Malawi-College of Medicine Research Support Centre in May 2016. This was obtained after a 3 days course.

- Certificate in Essential Burns care, obtained at Queen Elizabeth Central Hospital after a 3 days training, facilitated by the 'inter-burns' team from Glasgow in February 2015. This was obtained after a 2-day course and clinical sessions

4. PUBLICATIONS

a) As a 5th author, we (M'kumbuzi, Myezwa, Shumba and Namanja) contributed to chapter 1 of a book titled 'Knowledge for a sustainable world: A Southern African - Nordic contribution'. The chapter is titled 'Disability in southern Africa: Insights into its magnitude and nature'. This was published by African minds in 2015.

b) I have submitted a manuscript titled 'Impact of Cardiac rehabilitation treatment modalities in Sub-Saharan Africa: A systematic review' for possible publication in a Pan African Medical Journal, a peer review journal. This is awaiting reviewer's feedback.

c) I have submitted a manuscript titled 'cardiac rehabilitation; A potential management strategy for cardiovascular disease in Sub-Saharan Africa' for possible publication in a Pan African Medical Journal, and it is under reviewers' scrutiny.

d) I have submitted a manuscript titled 'quality of life of primary care givers of children with Cerebral Palsy in Blantyre', in Malawi Medical Journal; awaiting feedback.

5. EMPLOYMENT Lecturer, University of Malawi, College of Medicine in Blantyre (2019-present)

- Preparing lectures and teaching undergraduate physiotherapy students

- Servicing, as assigned by the head of department, other departments by offering lectures to their students

- Supervising physiotherapy students in their clinical work

- Mentoring physiotherapy students

- Contributing to evidence-based practice through conducting research

- Serving communities through practicing as a physiotherapist and health promoter in the teaching hospitals, clinics and communities

- Serving on committees as per assigned by the employer or representative
- Setting examinations and assessing students

Assistant lecturer, University of Malawi, College of Medicine in Blantyre (2015-2019)

- Preparing lectures and teaching undergraduate physiotherapy students under supervision
- Supervising physiotherapy students in their clinical work
- Mentoring Physiotherapy students
- Contributing to evidence-based practice through conducting research
- Serving communities through practicing as a physiotherapist and health promoter in the teaching hospitals, clinics and communities
- Serving on committees as per assigned by the employer or representative ➢ Assisting in setting examinations and assessing students

Physiotherapist intern, Queen Elizabeth Central Hospital in Blantyre (2014-2015)

- Assessing and treating patients with physiotherapy needs
- Serving communities through health promotion and prevention outreach programs and home visits for patients as necessary
- Supervising lower-cadre rehabilitation workers
- Supervising physiotherapy and rehabilitation technician students during their clinical placement at this hospital
- Managing the rehabilitation sections as per assigned by the head of department

Part -time Physiotherapist, Rehabilitation Hope in Blantyre Malawi (2014)

- Assessing and treating patients and clients requiring physiotherapy services
- Compiling monthly statistics for patients seen during that period and reporting to the managers

➢

Part-time research assistant, University of Malawi, College of Medicine in Blantyre (20132014)

I worked for two projects titled 'Impact of results-based financing on effective obstetric care coverage' and 'Performance Based Financing for maternal and neonatal health care services in Malawi.

Enrolling participants and administering consent forms

➢ Conducting interviews and collecting data using tablets

➢ Conducting observations during birth until last stage of labor and collecting data using checklist

➢ Conducting inventory checks of maternity wings and collecting data using checklist

➢ Sending the data collected by the electronic devices to the main server soon after data collection

➢ Sending the collected data by paper through sealed boxes to the main study office on weekly basis

Teacher, Limbikani Private Secondary School (2008-2009)

➢ Teaching mathematics and physical science to senior students ➢ Setting and marking examinations

➢ Discharging any other duty as per assigned by the head master

6. RESEARCH SKILLS

➢ Designing data collection tools

➢ Collecting quantitative and qualitative data

➢ Analyzing quantitative data using Epi info. And SPSS statistical packages ➢ Writing research report

7. CONFERENCES AND PRESENTATIONS

➢ Impact evaluation for sustainable development goals. This was a one-day conference organized by the Africa diaspora academic network at University of Central Lancashire in July 2019. I presented my proposed plan for developmental activities I would like to implement, through assistance of my employer, at my work place for the public. This

-
  presentation was delivered to different scholars from all fields of study and experts in developmental studies.
- Life after postgraduate studies: contributing towards development conference, organized by the commonwealth scholars in North-west region of England in April 2019.

- Maximizing your impact: training for development conference organized by the Commonwealth Scholarship Commission in March 15-17, 2019.
  Care for the elderly; survey of geriatric training for health care professionals in Malawi. I presented literature review findings for this topic to my colleagues and stakeholder organizations for older persons and geriatric care in Malawi, in October 2017

- Research dissemination conferences organized by University of Malawi, College of Medicine in the years 2015-2017.
- Better health for older person's conference at Ramada Resort Inn in Dar Es Salaam in Tanzania, organized by Age international (May 25-26, 2017). I presented the experiences of older people on health care accessibility and utilization in Malawi. There were over 10 countries (8 from Southern Africa and two from Europe) and different specialists working together to promote quality of lives for all senior members.

8. AWARDS

- Commonwealth scholarship for 2018-2019 master's degree at University of Chester, United Kingdom

- Small research grant offered by the office of postgraduate dean at College of Medicine, Malawi (2018)

- The most hardworking outgoing undergraduate student at University of MalawiCollege of Medicine in 2014

- Medic to Medic studentship in 2010

- The best student in mathematics and physical science at Providence girls' secondary school in 2008

9. PROFESSIONAL ASSOCIATIONS AND REGISTRATION

- Registered physiotherapist with Medical Council of Malawi since 2014

- Member of Physiotherapy Association of Malawi since 2012

-

10. VOLUNTARY WORK EXPERIENCE Health programmes coordinator, Malawi Network for older persons organisation (20162018)

- Representing the organisation in all forums involving care for older persons in Malawi
- Liaising with district health officers on the need to assist disadvantaged older persons in specific areas when need arises
- Advocating for specialised geriatric care services in Malawi
- I also was the first to initiate and establish the network between the organisation and University of Malawi-College of Medicine; the two are now working towards lobbying

for a geriatric department at the teaching hospital and they are also discussing on the need to establishing a geriatric care course

Rehabilitation officer, Lazarus group of hope Cameroon-Malawi Branch in Zomba district (2017-2018)

- Conducting needs assessment for people living with disabilities, orphans and single parent women
- Establishing the connections with orphanages, social workers and vocational training schools
- Referring people with physical needs for physiotherapy, occupation therapy or orthotic care in various hospitals

Physiotherapist, Karibu Geriatric Clinic in Blantyre Malawi (2017-2018)

- Assessing and offering physiotherapy services to older persons
- Documenting care delivery
- Liaising with the clinician in charge and the manager on the need for appropriate referrals when the need arises

Health advocator and physiotherapist, Malawi Medical Outreach Mission (2012-present)

- Preparing and delivering health talks on communicable and noncommunicable disease prevention and management
- Assessing and offering physiotherapy service to patients on palliative care in their homes
- Making appropriate referrals for further comprehensive physiotherapy or medical care service
- Cheering up the sick and the poor and giving them hope

11. REFEREES

Prof. Stephen Fallows
Postgraduate Research Coordinator
Department of Clinical Sciences and Nutrition
University of Chester
CH1 4BJ
Parkgate Road
Chester
Mob: +441244 513407
Email: s.fallows@chester.ac.uk

Mr. Chisati
Head of Physiotherapy Department
College of Medicine
Private Bag 360
Chichiri, Blantyre 3.
Mob: +265888168284
Email: echisati@medcol.mw
