# Supplementary material for: Delivering effective, comprehensive, multi-exercise component cardiac rehabilitation (CR) for chronic heart failure patients in low resource settings in sub-Saharan Africa: Queen Elizabeth Central Hospital—(QECH-CR) randomised CR study, Malawi
Source: PLoS One. 2024 May 24;19(5):e0297564. doi: 10.1371/journal.pone.0297564 (PMC11125511; doi:10.1371/journal.pone.0297564)
Supplement: Attachment — Submitted filename: CRP RCT at Malawi .docx [file pone.0297564.s004.docx]

Dear Authors,

Thank you very much for the opportunity to review your work on “Delivering effective, comprehensive, multi-exercise component cardiac rehabilitation (CR) for chronic heart failure patients in low resource settings in sub-Saharan Africa: Queen Elizabeth Central Hospital – RCT study, Malawi.” This is an RCT study of cardiac rehabilitation implemented on a novel hybrid exercise training model vs conventional usual care. A total of n=60 were recruited from baseline, and eventually, n=50 completed all data points at the end of 24 weeks.

I wish to congratulate the authors for their contributions to the field. However, I have some feedback/concerns before the manuscript should be considered ready for publication.

Introduction:

Page 4 line 90-95: Please standardise reference styles used. Also, a few unidentified symbols should be corrected before submission.

Page 5 line 102: Please clarify the abbreviations for QE CR, which I presume stand for Queen Elizabeth and cardiac rehabilitation; it is probably a good idea to provide the full terms before abbreviations are used.

Methods:

Page 5, lines 108, 109, and 114. Similar to those mentioned above, please provide the full term for QECH, UMIN-CTR, and BACPR.

Page 7, line 147: (r)elated data.

Page 7, line 169: I suggest adding a quick line of explanation as to why a 20-m long corridor was used for 6MWT, while ATS (2003) recommended a minimum of 25/30 metres. Limitations happen in clinical settings; maybe a short note to close the loop for the benefit of future readers.

Furthermore, please elaborate if participants were required to undergo the 6MWT once or twice. The information is missing.

Page 8, lines 184-186: Incomplete statement

Page 8, lines 186-187: please further elaborate on how the 1RM testing was performed.

Page 9, line 195: maybe replace the word guardians with caregivers? Given that the participants are grown-up adults.

Methods in general: please elaborate on how the data/result corresponding to “healthcare workers’ reported challenges to delivering CR at QECH” were collected. The whole section is missing from the Methods.

Results:

What is the final statistical power with n=60? That differed from the original sample size calculation of n=80.

Figure 2 & 3: maybe suggest revising the legends of the graph from “pre-heart rate response” and “post-heart rate response” to “Pre-ET heart rate response” and “Post-ET heart rate response.” I found it confusing initially, uncertain if it refers to pre-post chest step test or pre-post exercise training (CR).

Discussion: it would be interesting if the authors could discuss the intermediate long term training effects of this novel hybrid CR, which is currently missing. I read that the improvement of 6MWD in the ET group improved by 103.59+/-48.19 metres (table 4). However, the comparison of the change in 6MWD during the maintenance phase 30.36+/-31.3 and 43.53+/-73.8 metres at 1- and 3-months follow-up respectively.
